# 14-3-3 zeta is a molecular target in guggulsterone induced apoptosis in Head and Neck cancer cells

**DOI:** 10.1186/1471-2407-10-655

**Published:** 2010-11-30

**Authors:** Muzafar A Macha, Ajay Matta, SS Chauhan, KW Michael Siu, Ranju Ralhan

**Affiliations:** 1 Department of Biochemistry, All India Institute of Medical Sciences, New Delhi-110029, India; 2 Department of Chemistry and Centre for Research In Mass Spectrometry, York University, 4700 Keele Street, Toronto, Ontario, Canada M3J 1P3; 3 Department of Chemistry and Centre for Research In Mass Spectrometry, York University, 4700 Keele Street, Toronto, Ontario, Canada M3J 1P3; 4 Joseph and Mildred Sonshine Family Centre for Head and Neck Diseases and Department of Otolaryngology - Head and Neck Surgery, Mount Sinai Hospital, 600 University Avenue, Toronto, Ontario, Canada M5G 1X5; 5 Alex and Simona Shnaider Laboratory of Molecular Oncology, Mount Sinai Hospital, Toronto, Ontario, Canada, M5G 1X5; 6 Department of Pathology and Laboratory Medicine, Mount Sinai Hospital, Toronto, Ontario, Canada, M5G 1X5; 7 Department of Otolaryngology-Head and Neck Surgery, University of Toronto, Toronto, Ontario, Canada M5G 2N2

## Abstract

**Background:**

The five-year survival rates for head and neck squamous cell carcinoma (HNSCC) patients are less than 50%, and the prognosis has not improved, despite advancements in standard multi-modality therapies. Hence major emphasis is being laid on identification of novel molecular targets and development of multi-targeted therapies. 14-3-3 zeta, a multifunctional phospho-serine/phospho-threonine binding protein, is emerging as an effector of pro-survival signaling by binding to several proteins involved in apoptosis (Bad, FKHRL1 and ASK1) and may serve as an appropriate target for head and neck cancer therapy. Herein, we determined effect of guggulsterone (GS), a farnesoid X receptor antagonist, on 14-3-3 zeta associated molecular pathways for abrogation of apoptosis in head and neck cancer cells.

**Methods:**

Head and neck cancer cells were treated with guggulsterone (GS). Effect of GS-treatment was evaluated using cell viability (MTT) assay and apoptosis was verified by annexin V, DNA fragmentation and M30 CytoDeath antibody assay. Mechanism of GS-induced apoptosis was determined by western blotting and co-IP assays using specific antibodies.

**Results:**

Using in vitro models of head and neck cancer, we showed 14-3-3 zeta as a key player regulating apoptosis in GS treated SCC4 cells. Treatment with GS releases BAD from the inhibitory action of 14-3-3 zeta in proliferating HNSCC cells by activating protein phosphatase 2A (PP2A). These events initiate the intrinsic mitochondrial pathway of apoptosis, as revealed by increased levels of cytochrome c in cytoplasmic extracts of GS-treated SCC4 cells. In addition, GS treatment significantly reduced the expression of anti-apoptotic proteins, Bcl-2, xIAP, Mcl1, survivin, cyclin D1 and c-myc, thus committing cells to apoptosis. These events were followed by activation of caspase 9, caspase 8 and caspase 3 leading to cleavage of its downstream target, poly-ADP-ribose phosphate (PARP).

**Conclusion:**

GS targets 14-3-3 zeta associated cellular pathways for reducing proliferation and inducing apoptosis in head and neck cancer cells, warranting its investigation for use in treatment of head and neck cancer.

## Background

Head and neck squamous cell carcinoma (HNSCC) is the sixth most common cancer in the U.S. and the fourth most prevalent cancer in men worldwide, accounting for over 500,000 new cases annually [1]. The 5-year survival rate is less than 50%, and the prognosis of advanced cases has not improved much over the past three decades [2,3]. Despite standard multi-modality therapeutic interventions, including surgery, radiation and/or chemo-radiotherapy, head and neck cancer patients have a substantial risk of developing second primary tumors, often attributed to "field cancerization" - molecular alterations arising due to chronic carcinogen exposure of the upper aerodigestive tract [4-6]. Moreover, the limited efficacy, lack of safety, and high cost of mono-targeted therapies including EGFR inhibitors, limit their use in head and neck cancer management [7-9]. Hence major emphasis is being laid on identification of novel molecular targets and development of multi-targeted therapies. Clinical development of agents that can delay onset and/or progression could significantly improve the management of head and neck cancer.

Guggulsterone (GS), [4, 17(20)-pregnadiene-3, 16-dione], obtained from the plant *Commiphora mukkul *is used for treatment of obesity, hyperlipidemia, atherosclerosis, diabetes and osteoarthritis [10-12]. Besides, GS has also been reported to induce apoptosis, suppress proliferation, invasion, angiogenesis and metastasis in a wide variety of human cancer cell lines, including acute myeloid leukemia, head and neck, prostate, lung, breast, colon and ovarian cancer [13-22]. Interestingly, normal human fibroblasts, non-transformed prostate and colon epithelial cell lines are relatively resistant to growth inhibition by GS in comparison to cancer cells [13,16,18]. Various mechanisms have been proposed to explain the anti-carcinogenic effects of GS, including inhibition of reactive oxygen species (ROS), suppression of inflammation and inhibition of nuclear receptors (farnesoid X receptors), transcription factors [nuclear factor kappa B (NFκB), signal transducer and activator of transcription 3 (STAT3)], anti-apoptotic (Bcl-2, Bax, Bad and xIAP) and cell cycle-regulatory proteins (p21, p16 and cyclin D1). In addition, Leeman-Neill et al., [23] recently showed GS-treatment decreased the expression of both pSTAT3 (p-tyr-705), total STAT3 and hypoxia-inducible factor (HIF)-1α in HNSCC cell lines and in a xenograft model of HNSCC. Similarly, in our earlier reports, we also demonstrated GS reduced the levels of pSTAT3 (p-tyr-705) in both multiple myeloma and HNSCC cell lines [24].

14-3-3 family of proteins consists of seven members (ζ, σ, β, η, θ, γ and ε) which are multifunctional phospho-serine/phospho-threonine binding molecules that can serve as effectors of survival signaling [25]. Recently, using quantitative proteomics screens we identified a panel of proteins including 14-3-3 zeta, as biomarkers for diagnosis and prognosis of head and neck cancer with a high sensitivity and specificity [25-29] and suggested their involvement in development and progression of head and neck cancer. An emerging role for 14-3-3 zeta as an effectors of pro-survival signaling is suggested in part by the large number of 14-3-3 binding proteins involved in apoptosis, such as A20, ASK1, FKHRL1 and Bad [25,30-35]. In addition, using in vitro models of head and neck cancer, we also showed knocking down expression of 14-3-3 zeta sensitizes head and neck cancer cells to chemotherapy [36], revealing its therapeutic potential.

In the current study, we investigated the effect of GS on 14-3-3 zeta and its role in GS-induced apoptosis in HNSCC cells. Our results demonstrated GS-induces apoptosis by intrinsic mitochondrial pathway by a protein phosphatase mediated dephosphorylation of Bad, thus releasing it from inhibitory action of 14-3-3 zeta, which sequesters it in cytoplasm of proliferating cells.

## Methods

### Antibodies and Reagents

Z-guggulsterone (GS), 3-(4, 5-dimethylthiazol-2-yl)-2, 5-diphenyltetrazolium bromide (MTT), propidium iodide (PI) were purchased from Sigma (St. Louis, MO). For details of the antibodies used in this study, see Additional file 1: Supplementary Table S1. Caspase-3 activity assay kit was obtained from R&D systems (Minneapolis, MN); Caspase-9 and Caspase-8 activity assay kit from Genentech (South San Francisco, CA). Protein A-sepharose beads were obtained from GE Healthcare Biosciences, (Uppsala, Sweden).

### Cell culture

Human head and neck squamous carcinoma cell lines, SCC4 was obtained from American Type Culture Collection (ATCC) and HSC2 (JCRB0622) was obtained from Health Science Research Resources Bank (HSRRB), Japan, were grown in monolayer cultures in Dulbecco's modified eagle medium (DMEM) (Sigma, St. Louis, MO) supplemented with 10% fetal bovine serum (FBS) (Sigma), 1 mM L-glutamine, 1 mM minimum essential medium (MEM), 100 μg/ml streptomycin and 100 U/ml penicillin in a humidified incubator (5% carbon-dioxide, 95% air) at 37°C as described earlier [36]. For the assays described below, unless specified, head and neck cancer cells were cultured in 100 mm culture dishes for 24 h, followed by treatment with GS (50 μM) in DMEM only (without FBS) or medium containing DMSO (< 0.05%) which served as a vehicle control.

### Cell viability assay

Head and neck cancer cells (5 × 10^3^/well) were plated in a 96-well plate for 24 hrs. Cells were incubated in triplicates in the presence of medium containing GS (0 - 100 μM) or 0.02% of DMSO which served as a vehicle control in a final volume of 100 μl for 24 - 72 hrs at 37°C. Cell death was measured by adding 3-(4,5-dimethylthiazol-2-yl)-2,5-diphenyltetrazolium bromide (MTT) at 37°C for 3-4 h. The formazan crystals were dissolved in 100 μl of dimethylsulphoxide (DMSO) and optical density (OD) was measured at wavelength of 570 nm. The percentage cell death was calculated individually for each dose as follows: (OD_control _- OD_treated_/OD_control_) × 100. The percentage cell viability was calculated as [% of viable cells = (100 - % cell death)] [36].

### Cell cycle analysis using flow cytometry

GS-treated and untreated, control SCC4 cells collected at 24 h and 48 h were washed with phosphate buffer saline (PBS, pH = 7.4). The cells were fixed in 70% ethanol for 30 min at -20°C and resuspended in buffer containing PBS-EDTA (0.5 M, pH = 8.0), Triton X-100 (0.05%), RNAse A (50 μg/ml) and propidium iodide (PI, 100 μg/ml) for flow cytometry analysis. The PI-labeled cells were analyzed using a BD Canto flow cytometer and the output thus obtained was analyzed using the BD FACS Diva software (BD Biosciences, CA) [36].

### Annexin V assay

Annexin V and propidium iodide double staining was used to quantify apoptosis. Control and GS-treated SCC4 cells were harvested at 24 h and 48 h, washed with ice-cold PBS (pH = 7.4), resuspended in cold calcium binding medium (10 mM HEPES, pH 7.4, 140 mM NaCl and 2.5 mM CaCl_2_), and stained with FITC-labeled annexin V and propidium iodide, following manufacturer's protocol (R&D Systems, Minneapolis, MN). Following incubation at room temperature for 15 min in the dark, cells were analyzed with FACS CantoTM flow cytometer (BD Biosciences, San Jose, CA) [36].

### DNA fragmentation assay

SCC4 cells (2 × 10^6^) were treated with GS for different time intervals; harvested, resuspended in 200 μl of ice cold lysis buffer containing 10 mM Tris-HCl (pH = 7.4), 150 mM NaCl, 5 mM EDTA and 0.5% Triton X-100 and incubated for 30 min. Lysates were vortexed and cleared by centrifugation at 10,000 g for 20 min. Fragmented DNA in the supernatant was extracted with an equal volume of neutral phenol: choloroform: isoamyl alcohol (25:24:1, vol/vol/vol) and analyzed electrophoretically on 1.5% agarose gel containing ethidium bromide after measuring the DNA content using UV-spectrophotometer.

### M30 CytoDeath Antibody Assay

The M30 CytoDeath antibody binds to a caspase-cleaved formalin resistant epitope of cytokeratin 18, which is exposed during early apoptosis. Expression and localization of M30 protein was observed in GS treated and untreated control SCC4 cells using confocal laser scan microscopy as described by earlier [37]. Briefly, GS treated and control SCC4 cells grown over coverslip, were fixed in methanol for 20 min at -20°C, rinsed with PBS (pH = 7.2) and incubated with M30 CytoDeath antibody overnight at 4°C. After rinsing in PBS, the coverslips were incubated with biotinylated secondary antibody (LSAB plus Kit, DAKO Cytomations, Denmark) for 45 min at 37°C followed by incubation with streptavidin-conjugated fluorochrome, fluorescein isothiocyanate (FITC) (DAKO Cytomations, Glostrup, Denmark). Thereafter, the coverslips were counterstained with propidium iodide (PI, 10 mg/ml; Sigma, MO) for 30 sec. Coverslips were rinsed, mounted with antifade agent and examined using a confocal laser scanning microscope (CLSM)-LSM510 scanning module (Carl Zeiss Microscopy, Jena GmbH, Germany).

### Co-immunoprecipitation (co-IP) assays

Co-immunoprecipitation (co-IP) assays were carried out using specific antibodies and analyzed by western blotting. Briefly, head and neck cancer cells (SCC4) were rinsed in ice-cold PBS and lysed in IP lysis buffer [37]. Lysates were incubated on ice for 30 min. and cell debris was removed by centrifugation. Lysates were pre-cleared by adding 20 μl of Protein A Sepharose (GE Healthcare Biosciences, Uppsala, Sweden), followed by overnight incubation with specific antibodies (polyclonal anti-14-3-3zeta/anti-Bax or monoclonal anti-Bad/anti-Bcl2/anti-Bcl-xL antibody) on a rocker at 4°C. Immunocomplexes were pulled down by incubating with Protein A-Sepharose for 2 h at 4°C, followed by washing with 4X ice-cold lysis buffer to eliminate non-specific interactions [37]. Protein A-sepharose-bound immunocomplexes were then resuspended in Laemelli sample buffer, boiled for 5 min. and analyzed by Western blotting using specific antibodies as described below.

### Western blotting (WB)

Whole-cell lysates were prepared from GS-treated SCC4 cells and protein concentration was determined using the Bradford reagent (Sigma) and equal amounts of proteins (60 μg/lane) were resolved on 12% sodium dodecyl sulfate (SDS)-polyacrylamide gel [37]. The proteins were then electro-transferred onto polyvinylidene fluoride (PVDF) membrane. After blocking with 5% non-fat milk in Tris-buffered saline (TBS, 0.1 M, pH = 7.4), blots were incubated with specific antibodies as per manufacturer's recommended protocol at 4°C overnight. Protein abundance of α-tubulin (Santa Cruz Biotechnology, CA) served as a control for protein loading in each lane. Membranes were incubated with HRP-conjugated secondary antibodies, (DAKO Cytomation, Glostrup, Denmark), diluted at an appropriate dilution in 1% BSA, for 2 h at room temperature. After each step, blots were washed three times with Tween (0.1%)-Tris-buffer saline (TTBS). Protein bands were detected by the enhanced chemiluminescence method (ECL), Santa Cruz Biotechnology (Santa Cruz, CA) on XO-MAT film.

### Cytochrome c release Assay

SCC4 cells (2 × 10^5^/well) were cultured in 6-well plates for 24 h followed by treatment with GS washed with ice - cold PBS (pH = 7.2) and resuspended in isotonic mitochondrial buffer (210 mM mannitol, 70 mM sucrose, 1 mM EGTA, 10 mM Hepes, pH 7.5, 0.1% BSA) containing protease inhibitor cocktail. The resuspended cells were homogenized with a polytron homogenizer and then centrifuged at 2000 g for 3 min to pellet the nuclei and unbroken cells. The supernatant was centrifuged at 13,000 g for 10 min to pellet mitochondria [38]. The supernatant was further centrifuged at 15,000 g to pellet light membranes. The resulting supernatant contained the cytosolic fractions. The mitochondrial fraction was washed with mitochondrial buffer twice, resuspended with 1% Non-Idet P-40 lysis buffer, mixed for 60 min, and then centrifuged at 13,000 g for 10 min at 4°C. The supernatant containing mitochondrial proteins was collected and each sub-cellular fraction was subjected to Western blotting as described above.

### Caspase activity assay

Briefly, SCC4 cells (1 × 10^6^) were treated with GS for 0, 6, 12, 24 and 36 h at 37°C. The caspase enzyme activity in the cell lysates (treated and control) was measured by direct assay using synthetic fluorogenic substrate (Ac-DEVD-AFC, substrate for caspase 3 (R&D Systems) and Ac-LEHD-AFC, substrate for caspase 8 and caspase 9. The amount of fluorogenic 7-amino-4-methyl coumarin (AMC)/7-amino-4-trifluoromethyl coumarin (AFC) moiety released was measured using a spectofluorimeter.

### CD95/Fas assay

Briefly, SCC4 cells (1 × 10^6^) were treated with GS for 0, 6, 12, 24 and 36 h at 37°C. Cells were harvested, washed with PBS and incubated with FITC-conjugated antibody against CD95 (Fas) for 1 h at 4°C. After incubation, cells were washed, suspended in PBS containing 0.05% (w/v) sodium azide and analyzed using BD Canto flow cytometer and data were analyzed using the BD FACS Diva software. The gates were set to exclude dead cells and 10,000 cells were analyzed. Data are displayed as percentage of cells showing CD95 positivity.

### Statistical Analysis

Statistical analysis was carried out using SPSS 10.0 software (Chicago). Statistical significance was determined using the paired, two-tailed Student's t-test. p ≤ 0.05 was considered to be significant. Each assay was repeated at least 3 times (unless otherwise specified), to check the validity of the results obtained. The mean values ± standard deviations (S.D.) are presented in figures.

## Results

### GS inhibits cell proliferation and induces cell death

**Figure 1:**
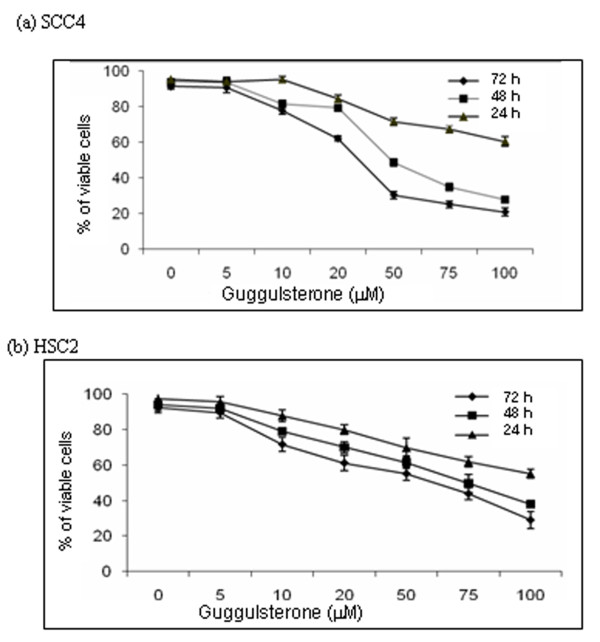
**GS reduces cell viability of HNSCC cell lines**. Cell viability was determined in SCC4 and HSC2 cells after 24 h - 72 h of incubation with 5, 10, 20, 50, 75 and 100 μM GS. Viable cell number was assayed by MTT uptake. Graphs show % of viable (**a**) SCC4 and (**b**) HSC2 cells on treatment with GS in a dose- and time-dependent manner. Each data point represents the mean ± standard deviation (S.D.) of 3 independent experiments.

**Figure 2:**
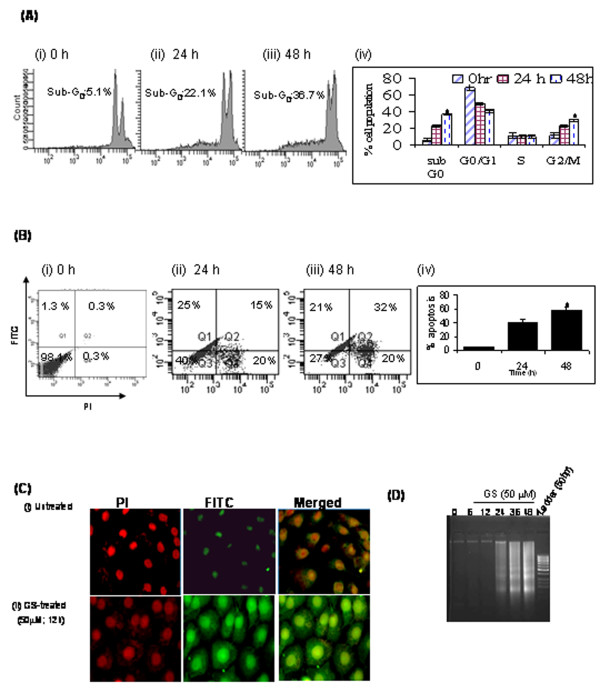
**GS induces cell death in HNSCC cells**. (**A**) SCC4 cells treated with 50 μM GS for 0 h, 24 h, and 48 h were fixed and stained with propidium iodide and cell cycle analysis was carried out using flow cytometry as described in Materials and Methods. Panel shows representative DNA histograms of GS-treated SCC4 cells in sub-G_0_, G_0_/G_1_, S- and G_2_/M phase of cell cycle after (i) 0 h, (ii) 24 h; (iii) 48 h; (iv) histograms showing mean % of cell population in each phase of cell cycle (* p < 0.05). (**B**) Annexin V assay. The panel shows % apoptotic cells on treatment with GS (50 μM) for (i) 0 h, (ii) 24 h and (iii) 48 h and (iv) histogram showing mean % of apoptotic cells (mean ± S.D., * p < 0.005). (**C**) Immunofluorescence analysis revealing M30 protein expression in SCC4 cells (i) untreated control cells; (ii) GS (50 μM) treatment for 12 h (original magnification × 400). (**D**) SCC4 cells were treated with GS (50 μM) for 6 h - 48 h followed by DNA extraction as described in materials and methods. Panel represents an agarose gel electrophoretogram showing DNA ladder confirming apoptosis.

To determine the cytotoxic effect of GS, head and neck cancer cells (SCC4/HSC2) were treated with varying concentrations (5 - 100 μM) for 24 h - 72 h. MTT assay revealed dose and time dependent increased cytotoxicity of GS in head and neck cancer cells (SCC4 and HSC2) as shown in Figure 1a and 1b respectively. Treatment of SCC4 cells with GS produced a cytotoxic effect with an inhibitory concentration at 50% (IC_50_) of 50 μM and 75 μM in SCC4 and HSC2 cells respectively. Therefore, 50 μM GS was used for all subsequent experiments in SCC4 cells. Flow cytometry analysis using propidium iodide staining showed significant increase in sub-G_0 _fraction of cell cycle in 24 h - 48 h indicating cell death in GS (50 μM) - treated SCC4 cells in comparison with untreated control cells (Figure 2A). Interestingly, GS-treated SCC4 cells also showed marked differences in G_2_/M fraction of cell cycle in comparison to untreated controls, as revealed by flow cytometry analysis (Figure 2A(iv).

### GS treatment induces apoptosis in head and neck cancer cells

Annexin V assay was carried out to evaluate GS induced apoptosis in head and neck cancer cells. GS treated SCC4 cells showed significant increase in apoptosis (40%) as early as in 24 h (Figure 2B). These results were further confirmed using M30 cyto-death and DNA fragmentation assay. Increased cytoplasmic expression of M30 protein and DNA fragmentation that are characteristics of apoptosis were observed in GS - treated SCC4 cells in 48 h (Figure 2C &2D). However, no detectable immunostaining was observed by M30-cytodeath assay in the control cells treated with vehicle alone, confirming induction of apoptosis by GS treatment (Figure 2C).

### Effect of GS on cell cycle regulatory and anti-apoptotic proteins

**Figure 3:**
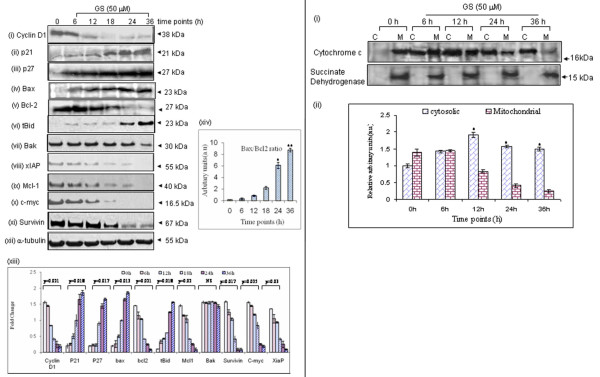
**(A) GS modulates cell cycle regulatory and anti-apoptotic proteins**. SCC4 cells were incubated for different time points (0 h - 36 h) with of GS (50 μM) and whole-cell extracts were prepared. Panel shows western blot analysis of (i) cyclin D1, (ii) p27, (iii) p21, (iv) Bax, (v) Bcl2, (vi) Bak, (vii) tBid, (viii) xIAP, (ix) Mcl1, (x) c-myc and (xi) survivin (xii) α-tubulin served as a loading control, (xiii) histograms showing fold change (mean ± S.D.) of protein expression, (xiv) histograms showing increased Bax/Bcl2 ratio (mean ± S.D.) in SCC4 cells (*^,^**p < 0.005) (**B**) Panel shows increased expression of cytochrome c in cytosolic fractions of GS-treated SCC4 cells in a time dependent manner (0 h - 36 h). Absence of succinate dehydrogenase (mitochondrial protein) expression in cytosolic fractions (C) confirmed the purity of cytosolic (C) and mitochondrial (M) sub-cellular fractions (* p < 0.05).

Treatment with GS (50 μM) decreased expression of cell cycle regulatory protein-cyclin D1, while it induced expression of cyclin-dependent kinase inhibitor p21^WAF1/CIP1 ^and p27 in SCC4 cells in a time dependent manner (Figure 3A(i-iii). Interestingly, GS treatment also induced a significant shift in the Bax/Bcl2 ratio within 36 h of GS treatment in SCC4 cells (Figure 3A(iv-v, xii), while it did not show marked effect on expression of Bak at the indicated time points (Figure 3A(vii), suggesting GS-induced apoptosis in head and neck cancer cells involves the intrinsic mitochondrial pathway. Treatment with GS (50 μM) suppressed the expression of anti-apoptotic proteins xIAP, Mcl1, c-myc and survivin in SCC4 cells (Figure 3A(viii-xi).

Cytochrome c release from mitochondria is the most important step in the apoptosis cascade involving intrinsic mitochondrial pathway. As shown in Figure 3B, we observed a significant increase in expression of cytochrome c protein in cytosolic fractions of SCC4 cells on treatment with GS in a time dependent manner.

### GS induces dissociation of Bad from 14-3-3 zeta to activate intrinsic mitochondrial pathway

**Figure 4:**
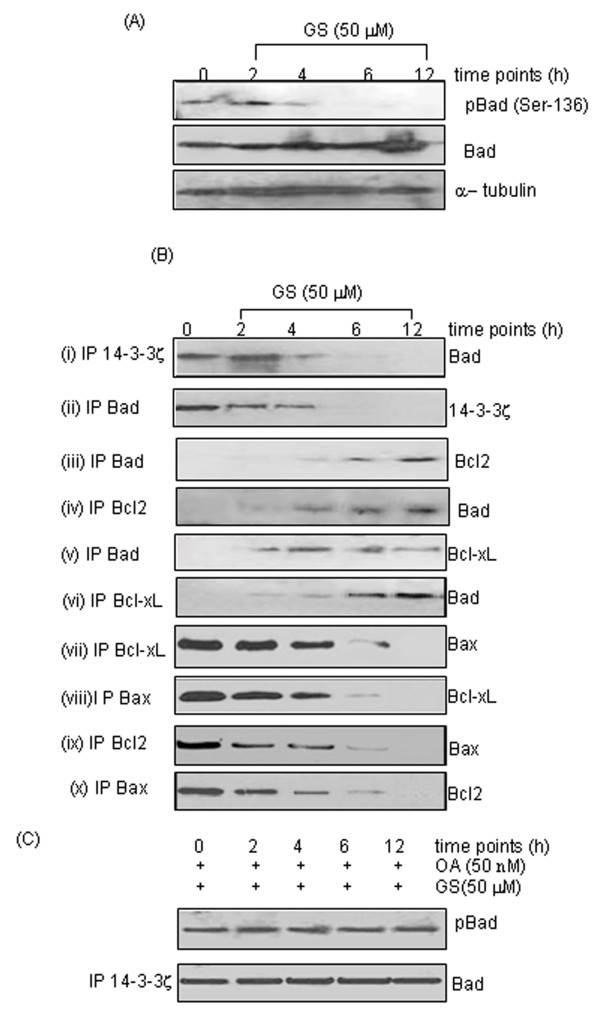
**(A) GS induces dephosphorylation of pBAD**. Whole cell lysates obtained from GS (50 μM) treated SCC4 cells were subjected to western blotting using specific antibodies for pBad, total Bad and α-tubulin. Panel shows the dephosphorylation of Bad in time-dependent manner (0 h - 12 h) while no difference was observed in the expression of total Bad protein in GS-treated SCC4 cells. α-tubulin served as loading control. (**B**) GS induces dissociation of Bad from 14-3-3ζ in head and neck cancer cells. Co-IP assays using specific antibodies were carried out and analyzed by western blotting as described in Materials and Methods. Treatment with GS induced dissociation of Bad from 14-3-3ζ as shown in panel (i) and (ii); association of Bad with Bcl2 as shown in panel (iii) and (iv); association of Bad with Bcl-xL (v) and (vi); and dissociation of pro-apoptotic protein Bax from Bcl-xL (vii and viii) as well as Bcl2 (ix and x) (**C**) Okadaic acid (OA) inhibits GS-induced dephosphorylation of Bad. Panel shows inhibition of dephosphorylation of Bad (Ser-136) and its dissociation from 14-3-3ζ in a time-dependent manner as determined using co-IP assays followed by western blotting.

The pro-apoptotic function of Bad requires its localization on outer mitochondrial membrane to release Bax from Bcl2/Bcl-xl, forming ion pores, thereby increasing membrane permeability releasing cytochrome c into cytoplasm. However, Bad is sequestered in cytoplasm by 14-3-3 zeta in proliferating cancer cells inhibiting its pro-apoptotic functions. Our results showed a time dependent decrease in expression of phosphorylated-Bad (pBad, Ser-136), while no significant change in expression of total Bad was observed on GS treatment in SCC4 cells (Figure 4A). Using co-immunoprecipitation (co-IP) assays, we showed that decrease in phosphorylation of Bad results in its dissociation from 14-3-3 zeta in the cytosol, on treatment with GS (50 μM) as shown in Figure 4B. The dephosphorylated Bad accumulates on outer mitochondrial membrane forming heterodimers with either Bcl2 or Bcl-xl releasing Bax from inhibitory action of these anti-apoptotic proteins as revealed by co-IP assays using specific antibodies (Figure 4B). These results were further confirmed by reverse co-IP assays followed by western blotting.

The dephosphorylation of Bad on Ser-136 has been reported by protein phosphatases 2A (PP2A) in response to various apoptotic stimulus [39]. Therefore, we evaluated dephosphorylation of Bad and its dissociation from 14-3-3 zeta on GS treatment (50 μM) in presence of serine/threonine phosphatase inhibitor, okadaic acid (OA). Our results clearly showed no change in dephosphorylation of pBad on treatment with GS in presence of okadaic acid (OA) (Figure 4C). Further, co-IP assays showed inhibition of dissociation of pBad from 14-3-3 zeta on GS-treatment in presence of OA, thereby inhibiting apoptosis (Figure 4C). These results suggest that GS recruits a phosphatase to release pro-apoptotic protein, Bad from 14-3-3 zeta activating the intrinsic mitochondrial pathway of apoptosis.

### Caspase assay

**Figure 5:**
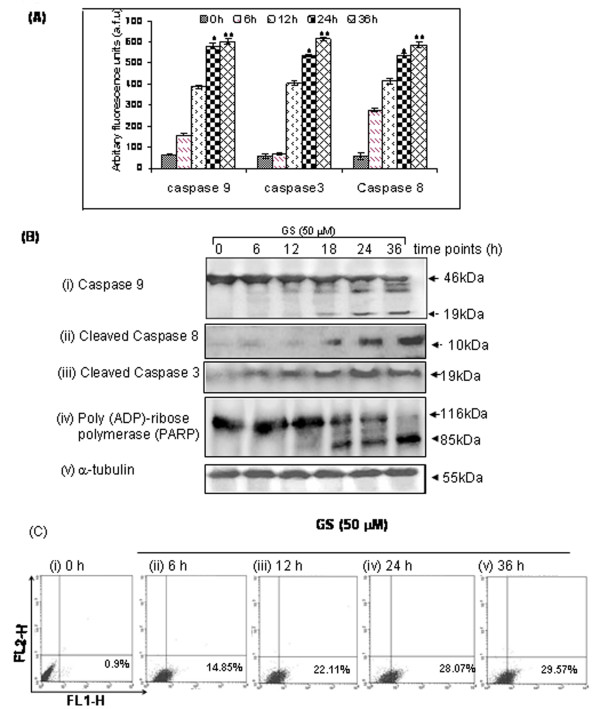
**(A) GS induces activation of caspases 8/9/3**. Quantitative fluorogenic assay for caspase activation was carried out as described in materials and methods. Treatment with GS (50 μM) induced significant increase in activation of caspases 8/9/3 (*^, ^** p < 0.05) in 6 h - 12 h as compared to vehicle control. (**B**) Western blot analysis showing (i) cleaved caspase 9; (ii) cleaved caspase 8; (iii) cleaved caspase 3; and (iv) cleaved PARP confirmed caspase-dependent apoptosis; (iv) α-tubulin served as loading control. (**C**) GS induced the expression of CD95/Fas. Panel shows % SCC4 cells expressing CD95/Fas receptor on treatment with GS (50 μM) (i) 0 h; (ii) 6 h; (iii) 12 h; (iv) 24 h; (v) 36 h using flow cytometry.

Caspase 9 and caspase 3 assays were carried out using fluorogenic substrates for quantitative analysis in GS-treated and untreated control SCC4 cells. Fluorimetric analysis revealed increased caspase 9 and caspase 3 activities in a time dependent manner with maximum activity observed at 36 h of GS treatment (Figure 5A). These results were further verified by presence of cleaved caspase 9 and caspase 3 using western blotting (Figure 5B). GS treatment resulted in cleavage of PARP starting from as early as 18 hrs into respective fragments of 116 kDa and 85 kDa with increased cleavage of PARP by 36 h (Figure 5B). Taken together, our results demonstrated GS induced apoptosis by activation of the mitochondrial pathway.

### Evaluation of extrinsic pathway/death receptor pathway in GS-induced apoptosis

In addition to induction of mitochondrial pathway for apoptosis, we also evaluated expression of Fas/CD95, truncated Bid (tBid), caspase 8 and its activity. Using flow cytometric analysis, we observed significant increase in Fas/CD95 expression (29.5%, p = 0.04) in SCC4 cells after 36 hrs with GS (Figure 5C). Western blot analysis showed increased expression of tBid (Figure 3A(vi) and cleaved caspase 8 (Figure 5B). These results were further supported by increased caspase 8 activity in GS treated SCC4 cells (Figure 5A), suggesting involvement of extrinsic pathways also in GS induced apoptosis.

## Discussion

Abrogation of apoptosis is important for survival and proliferation of cancer cells. Thus, targeting apoptosis is considered as an effective therapeutic strategy for treatment of cancer [37,38]. The Bcl2 family of proteins, including pro-survival members (Bcl2, Bcl-xL and Mcl1) and pro-apoptotic members (Bax, Bak and Bid) play key role in integrating apoptotic signals [39]. In addition, 14-3-3 proteins play an important role in regulating the balance between survival and apoptotic signaling in multiple ways in cancer cells. Kinases with pro-survival functions, such as AKT, Rsk and PIM, are responsible for generating the 14-3-3 docking sites on target proteins [25,30]. The binding of 14-3-3 frequently promotes re-localization of these pro-apoptotic proteins (Bad, Bax, FOXO and Ask1) away from their site of action [25,30-35]. The Bad-14-3-3 interaction causes Bad to be retained in the cytoplasm, thus, preventing Bad from dimerizing with Bcl2/xL at the mitochondria and mediating the release of Bax from Bcl2/xL-mediated inhibition [40]. Recently, we reported 14-3-3 zeta overexpression is associated with development, progression, poor prognosis and chemo-resistance in head and neck cancer cells and tissues [26-29]. These findings led us to propose the natural or chemical inhibitors targeting 14-3-3 zeta may serve as potential therapeutic agents for head and neck cancer.

**Figure 6:**
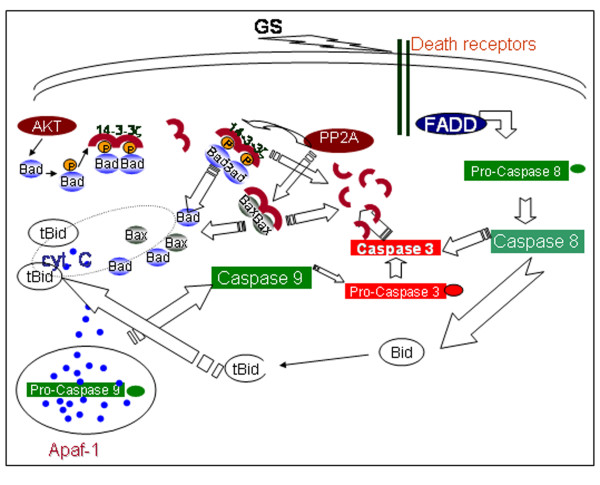
**The proposed model of GS-mediated apoptosis in HNSCC cells**. Our results demonstrated GS targets 14-3-3 zeta to initiate apoptosis through the intrinsic mitochondrial pathway by releasing Bad from its inhibitory action. Activation of PP2A inhibited association of Bad with 14-3-3 zeta resulting in its accumulation on outer mitochondrial membrane, altering its membrane potential. This releases cytochrome c, activating the downstream caspases - caspase 9 and caspase 3. In addition, treatment with GS induces expression of CD95/Fas receptors leading to caspase 8 activation in head and neck cancer cells.

GS is known anti-proliferative agent for inducing apoptosis in prostate, lung, colon and head and neck carcinomas, but the complete mechanism responsible for induction of apoptosis is unclear. In this study, using in vitro models of head and neck cancer, we showed 14-3-3 zeta as a key player regulating apoptosis in GS treated SCC4 cells. Our co-immunoprecipitation assays demonstrated that in proliferating, untreated control SCC4 cells, pBad (Ser 136) is sequestered in cytoplasm by 14-3-3 zeta, thereby abrogating apoptosis and promoting survival and proliferation. However, treatment with GS revealed dissociation of Bad from 14-3-3 zeta, leading to its accumulation on outer mitochondrial membrane in SCC4 cells. Here, Bad associates with Bcl2/xL, thereby, releasing Bax to form mitochondrial permeability transition pores in GS-treated SCC4 cells. Dissociation of Bad from 14-3-3 zeta and its association with Bcl2/xL, was verified by reverse immunoprecipitation assays. Further, we showed dissociation of Bad from 14-3-3 zeta was a result of dephosphorylation of pBad (Ser 136), a crucial step in decision making for induction of apoptosis. Interestingly, GS treatment activated PP2A phosphatases resulting in cytoplasmic accumulation of dephosphorylated Bad in SCC4 cells. In addition, changes in the Bax/Bcl2 ratio also affect mitochondrial membrane potential in cells [39,40]. Our results of western blotting showed increased Bax/Bcl2 ratio, altering the mitochondrial membrane potential in GS treated SCC4 cells. This results in leakage of cytochrome c from mitochondria into cytoplasm initiating apoptosis on treatment with GS. Another noteworthy observation in our study was significant decrease in expression of anti-apoptotic proteins, Bcl2, xIAP, Mcl1, survivin, cyclin D1 and c-myc on treatment with GS in comparison with no treatment controls, thus committing cells to apoptosis. These events were followed by activation of caspase 9 and caspase 3 resulting in cleavage of poly (ADP-ribose) polymerase (PARP) in GS treated SCC4 cells. Taken together, these observations suggested 14-3-3 zeta plays a key role in GS-induced apoptosis in head and neck cancer cells as shown in Figure 6.

Similar to our results, GS treatment has been shown to alter mitochondrial membrane potential releasing cytochrome c and initiating apoptosis leukemia, prostate and colon cancer [15,16,18]. GS also mediates its effects through downregulation of Akt pathway which promotes survival and proliferation, but activates JNK pathway inducing apoptosis [13]. In addition, our results also showed increase in expression of CD95/Fas upon GS treatment, in a time dependent manner. This increase in expression of death receptor CD95 coincides with the activation of caspase-8. GS-treatment increased expression of truncated Bid (tBid) in SCC4 cells, in a time dependent manner. Bid is a pro-apoptotic BH3-only member of the Bcl-2 family and an important component of Fas-induced apoptosis [41-43]. Bid is cleaved by active caspase-8 enhancing cytochrome c release from mitochondria into the cytosol. Thus, our findings suggest involvement of both 14-3-3 zeta dependent intrinsic and extrinsic pathway in GS induced apoptosis (Figure 6).

## Conclusion

In conclusion, we demonstrated guggulsterone as an effective cytotoxic agent inducing apoptosis in head and neck cancer cells, which are generally chemoresistant. Further, we showed GS targets 14-3-3 zeta to initiate apoptosis through intrinsic mitochondrial pathway by releasing Bad from its inhibitory action. Taken together, our results provide a biologic rationale for designing further studies investigating the clinical utility of GS as a potential complementary therapeutic/chemopreventive agent for head and neck cancer management.

## List of Abbreviations

HNSCC: Head and neck squamous cell carcinoma; MTT: 3-(4, 5-dimethylthiazol-2-yl)-2, 5-diphenyltetrazoliumbromide; PI: Propidium iodide; DMSO: Dimethylsulphoxide; FITC: Fluorescein isothiocyanate; AMC: 7-Amino-4-methyl coumarin; AFC: 7-Amino-4-trifluoromethyl coumarin

## Competing interests

The authors declare that they have no competing interests.

## Authors' contributions

MM carried out the experiments and contributed in preparing this manuscript. AM analyzed the experiments and contributed in preparing this manuscript. SSC and KWM Siu provided the financial support and edited the manuscript. RR conceived the idea and planned the experiments and contributed in preparing this manuscript. All authors read and approved the final manuscript.

## Pre-publication history

The pre-publication history for this paper can be accessed here:

http://www.biomedcentral.com/1471-2407/10/655/prepub

## Supplementary Material

Additional file 1 **Supplementary Table S1**. Details of the antibodies used in this study. Click here for file
