# Supplementary material for: 14-3-3 zeta is a molecular target in guggulsterone induced apoptosis in Head and Neck cancer cells
Source: BMC Cancer. 2010 Nov 30;10:655. doi: 10.1186/1471-2407-10-655 (PMC3002356; doi:10.1186/1471-2407-10-655)
Supplement: Additional file 1 — Supplementary Table S1. Details of the antibodies used in this study. [file 1471-2407-10-655-S1.DOC]

**Supplementary Table S1: Details of the antibodies**

| **S. No** | **Antibody** | **Catalogue #** | **Clone** |
| --- | --- | --- | --- |
| 1. | Mouse monoclonal Bad antibody | sc-8044 | C-7 |
| 2. | Goat polyclonal pBAD antibody | sc-7999 | Ser-136 |
| 3. | Rabbit polyclonal Bak antibody | sc-7873 | H-211 |
| 4. | Rabbit polyclonal Bax antibody | sc-493 | N-20 |
| 5. | Mouse monoclonal Bcl-xL antibody | sc-8392 | H-5 |
| 6. | Mouse monoclonal Bcl2 antibody | sc-493 | C-2 |
| 7. | Monoclonal cyclin D1 antibody | sc-8396 | A-12 |
| 8. | Rabbit polyclonal p21 antibody | sc-397 | C-19 |
| 9. | Rabbit polyclonal p27 antibody | sc-528 | C-19 |
| 10. | Mouse monoclonal anti-tubulin antibody | sc-5386 | B-7 |
| 11. | Mouse monoclonal Survivin antibody | sc-17779 | D-8 |
| 12. | Rabbit polyclonal Mcl1 antibody | sc-20679 | H-260 |
| 13. | Rabbit polyclonal 14-3-3ζ antibody | Sc-1019 | C-16 |
| 14. | Rabbit polyclonal xIAP antibody | Abcam | Ab21278 |
| 15. | FITC conjugated Fas antibody | BD Biosciences | 555673 |
| 16. | Caspase 9 | BD PharMingen | --- |
| 17. | Cleaved caspase 8 | Cell Signaling Technology | --- |
| 18. | Cleaved caspase 3 | Cell Signaling Technology | ---- |
